# COVID-19: Impact on radiology departments and implications for future service design, service delivery, and radiology education

**DOI:** 10.1259/bjr.20210632

**Published:** 2021-10-27

**Authors:** Alasdair Taylor, Craig Williams

**Affiliations:** 1 University Hospitals of Morecambe Bay NHS Foundation Trust, Royal Lancaster Infirmary, Lancaster, United Kingdom

## Abstract

The pandemic caused by SARS-CoV-2 (severe adult respiratory distress syndrome Coronavirus-2) and its most severe clinical syndrome, COVID-19, has dramatically impacted service delivery in many radiology departments. Radiology (primarily chest radiography and CT) has played a pivotal role in managing the pandemic in countries with well-developed healthcare systems, enabling early diagnosis, triage of patients likely to require intensive care and detection of arterial and venous thrombosis complicating the disease. We review the lessons learned during the early response to the pandemic, placing these in the wider context of the responsibility radiology departments have to mitigate the impact of hospital-acquired infection on clinical care and staff wellbeing. The potential long-term implications for design and delivery of radiology services are considered. The need to achieve effective social distancing and ensure continuity of service during the pandemic has brought about a step change in the implementation of virtual clinical team working, off-site radiology reporting and postgraduate education in radiology. The potential consequences of these developments for the nature of radiological practice and the education of current and future radiologists are discussed.

## SARS-CoV-2

The syndrome of pneumonia leading to respiratory failure now known as COVID-19 was first described in Wuhan, China, in November 2019. Within weeks the causative agent had been identified, assigned the name SARS-CoV-2,^1^ and its genetic sequence published. The virus is primarily spread by contaminated droplets or aerosols from the respiratory tract of infected persons and by fomites – virus-contaminated residues of desiccated droplets in the vicinity of an infected patient.^2^ Viable virus particles have also been recovered from the faeces of infected patients,^3^ but evidence of faecal-oral transmission is scanty.

SARS-CoV-2 is substantially more contagious than seasonal influenza (with which it is often compared).^4^ Maximal virus shedding by infected patients occurs in the prodromal or early symptomatic phases of infection, explaining the observed “super spreader” individuals and events. It also exhibits longer prodromal and convalescent periods during which patients may transmit the infection. Its propensity to cause severe infection and death (1–2% mortality among laboratory-confirmed cases) is also an order of magnitude greater than that of seasonal influenza.^5^

The risk posed by “aerosol-generating procedures” (AGPs) as a route of transmission has been of particular concern. There is no universal definition of an aerosol, but the concept is that of virus-contaminated droplets small enough to remain airborne for an extended period, spreading further than 2 m from the source.^6^ This results in contamination of a wide area and a requirement for enhanced decontamination protocols. It also mandates the use of facemasks or breathing apparatus capable of filtering particles small enough to penetrate standard surgical facemasks (EN149 FFP3 specification). Lists of AGPs published by public health agencies have focussed primarily on those causing increased airflow across the upper respiratory tract epithelium,^7^ including high-flow oxygen, mechanical ventilation and use of suction during dental surgery and endoscopy of the airways and upper GI tract. Colonoscopy and CT colonography have not been classified as AGPs, although a study published in this issue of BJR offers some evidence to the contrary.^8^ In a case control study comparing CT colonography with routine body CT, the authors found evidence of airborne spread of coliform bacteria in more than one-third of colonography examinations. The very limited evidence published to date suggests that the absolute risk of transmission posed by colonoscopy or colonography is extremely low,^9,10^ but there is a need for further, larger scale research into the epidemiology of SARS-CoV-2 transmission in the healthcare setting to mitigate future risk to health and avoidable disruption to healthcare delivery.^11^

### Early impacts of, and responses to, the pandemic

Initial containment efforts followed established guidelines and included isolation or cohorting of infected patients, physical distancing of patients and staff, use of personal protective equipment (PPE – facemasks, gloves, gowns and eye protection) and rigorous room and equipment decontamination.^12^ Hospitals in countries with prior experience of Coronavirus epidemics (SARS and MERS) appeared better prepared to control nosocomial spread of infection, having established protocols and better access to PPE, while large numbers of infections and deaths occurred among healthcare workers in countries experiencing a Coronavirus epidemic for the first time.^11^

Nosocomial outbreaks among clinical teams threatened the sustainability of services as a result of staff absence due to illness or self-isolation. The requirement to implement effective physical distancing, safe patient flows and effective decontamination of equipment in radiology departments with overcrowded, cramped and inadequately ventilated accommodation necessitated substantially reduced patient throughput. During the “first wave” of the pandemic, government-mandated curtailment of much out-patient activity and fear of nosocomial infection among patients resulted in a substantial reduction in cancer diagnosis and a rapid increase in those awaiting scheduled surgical treatment.

### Lessons learned and future implications

Much has been learned as a result of these early experiences. While the fundamental design and allocation of sufficient space to radiology departments is best achieved through strategic planning decisions, radiologists can collaborate with infection control specialists to influence the reconfiguration of existing space and siting of new equipment so as to facilitate the adoption of safely segregated workstreams, either on a permanent basis or as part of resilience planning for future events. The case for investment in new imaging equipment, particularly CT scanners and point-of-care radiography and ultrasound machines, can only be strengthened by recent events.

Efforts to contain SARS-CoV-2 have for the first time relied heavily on extensive testing of asymptomatic patients and healthcare staff. The adoption of new practices such as pre-appointment telephone screening of out-patients, reorganisation of departments and workflows and implementation of guidelines developed by the National Institute for Health and Care Excellence and specialist societies^13,14^ has facilitated the safe restoration of diagnostic services.^9,10^ We must redouble efforts to optimise patient scheduling and flow by harnessing the capabilities of radiology information systems and electronic patient records.^15^ This may include linking hospital records with evidence of vaccination status. Close attention must be paid to adequate provision of PPE and regular training in its correct use, as it is known to be highly effective in preventing cross-infection.^16^ Radiologists have a duty to advocate for safe patient care as healthcare systems attempt to recover lost activity; permitting excessive crowding of departments and unrealistic patient scheduling risks further outbreaks not just of COVID-19, but a range of hospital-acquired infections.

### Impacts on radiology education and practice

Early in the pandemic, it became clear that social distancing requirements meant that face-to-face clinical conferences and classroom-based radiology education could not continue. Video-conferencing, already established as a means of enabling decision-making across multiple sites in a clinical network, has been superseded at unprecedented pace by cheaper, secure web-based applications utilising existing computer infrastructure, ushering in the “virtual multidisciplinary meeting”. Within months, radiology training and continuing medical education providers have adapted or developed content to exploit these channels. Many hospitals have accelerated the roll-out of teleradiology solutions for radiologists in response to staff demands to work remotely and the potential for an outbreak of infection to disrupt service delivery.

These developments have met the immediate challenge of the pandemic, but their consequences for future radiological practice and education have yet to be felt. The ability to work from home, while attractive to those eager to achieve “life-work balance”, potentially diminishes the intangible contribution to patient care and job satisfaction arising from in-person interaction with clinical colleagues and may result in reduced influence within the hospital. Existing multidisciplinary teams have functioned effectively online during the emergency; it remains to be seen how well newcomers can integrate into teams or how dysfunction in existing teams is resolved.

The paradigm shift to online delivery of training and education has been welcomed by most trainees and practising radiologists. Challenges to the continuation of traditional conferences, with their attendant travel and accommodation costs, will undoubtedly follow. On the other hand, online content will undoubtedly continue to improve, and offers opportunities to “level up” access to the best educational content, as exemplified by Radiopaedia.org, which offers reduced-cost access to subscribers in low-income countries.

### Control of other infection hazards in the radiology department

While COVID-19 has naturally dominated recent thinking, it should not be forgotten that radiology departments have a shared responsibility to control many other microbiological hazards to patients and staff. The morbidity, mortality and costs of hospital-acquired infections have grown substantially in recent years. This is a multifactorial problem: causes include the emergence of multidrug resistant bacteria, increasing numbers of patients with impaired host defences as a result of cytotoxic and immunomodulating drug therapies, and lapses in established infection prevention protocols. The modern radiology department has become a “meeting point” where it is all too easy for cross-infection to occur, and there is evidence that radiology department staff may lack the knowledge and training to implement effective infection control practices.^13^ Repeated reports of outbreaks of infection due to failure of basic infection control protocol relating to ultrasound gel exemplify this,^17^ but numerous studies have demonstrated the potential for infection to spread via contaminated machines, shared computer keyboards, but above all as a result of inadequate hand hygiene.^17,18^

The measures required to achieve control of most microbiological hazards are generally simple and effective if applied correctly.^19^ When faced with the global epidemic of human immunodeficiency virus (HIV) 35 years ago, healthcare staff rapidly embraced a set of “general measures” devised to prevent nosocomial transmission of HIV (then a uniformly fatal disease) and other bloodborne viruses.^20^ The COVID-19 pandemic has reminded us of the potentially devastating consequences of lapses in infection control practice. We must respond by embracing a culture in which driving down the incidence of healthcare-acquired infections is seen as both an organisational and a moral imperative. This will require increased investment in staff training, acceptance of personal responsibility and rigorous enforcement of infection control policy.
